# Bidirectional effect of vitamin D on brown adipogenesis of C3H10T1/2 fibroblast-like cells

**DOI:** 10.7717/peerj.14785

**Published:** 2023-01-31

**Authors:** Takako Mukai, Tatsuya Kusudo

**Affiliations:** Department of Nutrition and Food Sciences, Faculty of Human Sciences, Tezukayama Gakuin University, Sakai, Osaka, Japan

**Keywords:** Vitamin D, Brown adipocyte, BAT, C3H10T1/2, 3T3-L1

## Abstract

**Background:**

Brown adipose tissue (BAT) dissipates caloric energy as heat and plays a role in glucose and lipid metabolism. Therefore, augmentation and activation of BAT are the focus of new treatment strategies against obesity, a primary risk factor of metabolic syndrome. The vitamin D system plays a crucial role in mineral homeostasis, bone metabolism, and cell proliferation and differentiation. In this study, we investigated the effects of vitamin D_3_ [1,25(OH)_2_D_3_] on brown adipocyte differentiation.

**Methods:**

The mouse fibroblast-like cell line C3H10T1/2 was differentiated into brown adipocytes in the presence of 1,25(OH)_2_D_3_. The effect of 1,25(OH)_2_D_3_ on brown adipocyte differentiation was assessed by measuring lipid accumulation, the expression of related genes, and cytotoxicity. The viability of C3H10T1/2 cells was measured using the Cell Counting Kit-8 assay. Gene expression was investigated using quantitative reverse transcription-polymerase chain reaction. Protein expression was estimated using western blotting.

**Results:**

1,25(OH)_2_D_3_ inhibited adipocyte differentiation and exerted a cytotoxic effect at 1 nM. However, in the physiological concentration range (50–250 pM), 1,25(OH)_2_D_3_ promoted uncoupling protein 1 (UCP1) expression in C3H10T1/2 cells. This effect was not observed when 1,25(OH)_2_D_3_ was added 48 h after the initiation of differentiation, suggesting that the vitamin D system acts in the early phase of the differentiation program. We showed that 1,25(OH)_2_D_3_ increased the expression of two key regulators of brown adipogenesis, PR domain containing 16 (*Prdm16*) and peroxisome proliferator-activated receptor *γ* coactivator-1*α* (*Pgc1*α**). Furthermore, 1,25(OH)_2_D_3_ increased *Ucp1* expression in 3T3-L1 beige adipogenesis in a dose-dependent manner.

**Conclusion:**

These data indicate the potential of vitamin D and its analogs as therapeutics for the treatment of obesity and related metabolic diseases.

## Introduction

Obesity is a major risk factor of metabolic syndrome. The increasing prevalence of obesity has become a worldwide concern, and effective treatments for obesity-related diseases are of growing importance. The fundamental cause of obesity is an energy imbalance between calorie intake and calorie use, and adipose tissue plays an essential role in this process. In general, two types of adipose tissue, white (WAT) and brown adipose tissue (BAT), exist in mammals. WAT stores excess energy as triglycerides, whereas BAT dissipates energy as heat.

BAT specializes in thermogenesis and plays a crucial role in cold adaptation in small rodents by regulating nonshivering thermogenesis. Studies using mouse models have also shown that BAT has a regulatory role in glucose and lipid metabolism (Stanford et al., 2013; Bartelt et al., 2011). In humans, BAT was initially thought to exist at physiologically significant levels in newborns and to become essentially absent in adults. However, recent studies using positron emission tomography-computed tomography have shown that a physiologically significant amount of BAT exists in adults, and its presence is inversely related to body mass index and levels of visceral fat (Cypess et al., 2009; Saito et al., 2009; Virtanen et al., 2009). Moreover, BAT activation increases whole-body glucose disposal and insulin sensitivity in humans (Chondronikola et al., 2014; Orava et al., 2013). BAT also plays a significant role in human whole-body lipid metabolism (Chondronikola et al., 2016). Recent studies have demonstrated that the presence of BAT correlates with low odds of type 2 diabetes, dyslipidemia, coronary artery disease, cerebrovascular disease, congestive heart failure, and hypertension (Becher et al., 2021). Chronic cold stimulation or capsinoid intake can lead to the recruitment of BAT even in individuals with low or no detectable BAT activity (Yoneshiro et al., 2013). Consequently, BAT is emerging as a promising target for the treatment of obesity and related metabolic diseases (Cheng et al., 2021; Singh et al., 2021).

Vitamin D is well known for its role in the regulation of calcium and phosphate homeostasis. It also regulates proliferation, differentiation, and apoptosis in several cell lines (Nagpal, Na & Rathnachalam, 2005; Fleet et al., 2012) and is associated with several metabolic processes in the cardiovascular (Chen et al., 2011) and immune (Liu et al., 2006; Prietl et al., 2013) systems. Accumulating evidence indicates that vitamin D deficiency is associated with metabolic diseases (Park, Pichiah & Cha, 2018; Theik et al., 2021).

Several studies have examined the relationship between adipocyte differentiation and vitamin D (Vu et al., 1996; Kelly & Gimble, 1998; Blumberg et al., 2006; Kong & Li, 2006; Nimitphong et al., 2012; Basoli et al., 2017; Shi et al., 2002; Huang et al., 2002; Duque, Macoritto & Kremer, 2004; Zhuang, Lin & Yang, 2007; Cianferotti & Demay, 2007; Sun & Zemel, 2008; Sakuma et al., 2012; Ricciardi et al., 2015; Chang & Kim, 2016). In most studies, 1,25-dihydroxyvitamin D_3_ (1,25(OH)_2_D_3_), the most biologically active vitamin D_3_ metabolite, has been reported to have an inhibitory effect on adipogenesis. However, certain studies have reported a promoting effect of 1,25(OH)_2_D_3_ on adipogenesis. These discrepancies have been attributed to differences in the cell types and concentrations of 1,25(OH)_2_D_3_ used in these studies. Previously, 1,25(OH)_2_D_3_ was reported to have an inhibitory effect on brown adipocyte differentiation (Ricciardi et al., 2015). However, the concentration of 1,25(OH)_2_D_3_ used in that study was far higher than that typically observed in physiological settings. Consequently, the effects of physiological concentrations of 1,25(OH)_2_D_3_ on brown adipocyte differentiation remain ambiguous.

Therefore, we aimed to demonstrate that 1,25(OH)_2_D_3_ stimulates the brown adipocyte differentiation program at physiologically relevant concentrations. Our findings suggest that vitamin D may have therapeutic utility in the treatment of obesity and related metabolic diseases.

## Materials & Methods

### Cell culture and induction of adipogenesis

C3H10T1/2-clone 8 cells (#IF050415) were obtained from the Health Science Research Resources Bank (Osaka, Japan). Cells were grown at 37°C in a 5% CO_2_ humidified atmosphere in Dulbecco’s modified Eagle’s medium (DMEM) containing 10% fetal bovine serum (FBS). On reaching confluency, cells were induced to differentiate into brown adipocytes by supplementing the culture medium with 10 µg/mL insulin (Nacalai Tesque, Kyoto, Japan), 1 µM dexamethasone (DEX; Sigma-Aldrich, St. Louis, MO, USA), 0.5 mM 3-isobutyl-1-methylxanthine (IBMX; Sigma-Aldrich), 125 µM indomethacin (Cayman Chemical, Ann Arbor, MI, USA), and 3 nM 3,3,5-triiodo-L-thyronine (T3; Sigma-Aldrich). Two days after stimulation, the medium was replaced with DMEM supplemented with 10% FBS, 10 µg/mL insulin, and 3 nM T3 as previously described (Kusudo et al., 2019). Thereafter, the medium was changed every two days for a period of seven to eight days. Cells were treated with 1,25(OH)_2_D_3_ (Sigma-Aldrich) at the indicated doses depending on the experiment. 3T3-L1 cells (Japanese Collection of Research Bioresources Cell Bank, Tokyo, Japan) were stimulated for differentiation using DMEM, containing 10% FBS, 10 µg/ml insulin, 1 µM DEX, and 0.5 mM IBMX, for two days, followed by DMEM supplemented with 10% FBS and 10 µg/ml insulin. The medium was changed every two days for eight days. 1,25(OH)_2_D_3_ or ethanol was added to the media during the experiment. For beige adipocyte differentiation, 3T3-L1 cells were differentiated with DMEM, supplemented with 10% FBS, 10 µg/ml insulin, 1 µM DEX, and 0.5 mM IBMX, 10 µM rosiglitazone (Sigma-Aldrich), and 3 nM T3, for 48 h (Asano et al., 2014). Subsequently, the medium was replaced with DMEM containing 10% FBS, 10 µg/mL insulin, 10 µM rosiglitazone and 3 nM T3 every two days for eight days. The cells were stimulated with 10 µM isoproterenol for 4 h before harvesting.

### Vitamin D receptor (VDR) silencing in C3H10T1/2 cells

The VDR and negative control Stealth small interfering RNAs (siRNAs) (#MSS238646 and #12935200, respectively) were obtained from Thermo Fisher Scientific (Waltham, MA, USA). siRNAs were transfected into cells using Lipofectamine RNAiMAX reagent (Thermo Fisher Scientific) according to the manufacturer’s protocol. The following day, the medium was replaced with DMEM supplemented with 10% FBS. Three days after transfection, the medium was replaced with a differentiation medium for differentiation into brown adipocytes. In addition, to enhance UCP1 expression, C3H10T1/2 cells were stimulated with 1 µM all-trans retinoic acid (Sigma-Aldrich) for the last 24 h of differentiation.

### Quantitative reverse transcription-polymerase chain reaction (qRT-PCR)

Total RNA was extracted using TRI Reagent (Molecular Research Center Inc., Cincinnati, OH, USA), according to the manufacturer’s protocol. To quantify mRNA expression levels, qRT-PCR analysis was performed using a StepOne real-time PCR system (Applied Biosystems, Foster City, CA, USA) and PowerUp SYBR Green Master Mix (Thermo Fisher Scientific), as described previously (Kusudo et al., 2019). All gene expression data were normalized to the 36B4 expression levels. The respective sense and antisense oligonucleotide primers for the target genes were as follows: *Cebpa*, 5′-CAAGAACAGCAACGAGTACCG-3′ and 5′-GTCACTGGTCAACTCCAGCAC-3′; *Cebpb*, 5′-ACGACTTCCTCTCCGACCTCT-3′ and 5′-CGAGGCTCACGTAACCGTAGT-3′; *Cebpd*, 5′-CGACTTCAGCGCCTACATTGA-3′ and 5′-CTAGCGACAGACCCCACAC-3′; *Plin*, 5′-CTGTGTGCAATGCCTATGAGA-3′ and 5′-CTGGAGGGTATTGAAGAGCCG-3′; *Vdr*, 5′-GAATGTGCCTCGGATCTGTGG-3′ and 5′-ATGCGGCAATCTCCATTGAAG-3′; *Cox7a1*, 5′-AGAAAACCGTGTGGCAGAGA-3′ and 5′-CAGCGTCATGGTCAGTCTGT-3′; *Cox8b*, 5′-GCGAAGTTCACAGTGGTTCC-3′ and 5′-GAACCATGAAGCCAACGACT-3′; *MT-CO1*, 5′-CAAGAACAGCAACGAGTACCG-3′ and 5′-GTCACTGGTCAACTCCAGCAC-3′; and *Ndfvu1*, 5′-ACGACTTCCTCTCCGACCTCT-3′ and 5′-CGAGGCTCACGTAACCGTAGT-3′. All other oligonucleotide primer sets used in this study have been described previously (Kusudo et al., 2019; Hashimoto et al., 2019).

### Western blot analysis

Western blotting was performed as previously described (Mukai et al., 2017). Briefly, C3H10T1/2 cells were lysed with radioimmunoprecipitation assay buffer, and protein concentrations were determined using the Pierce BCA Protein Assay Kit (Thermo Fisher Scientific). Samples containing equal amounts of protein were separated by sodium dodecyl sulfate–polyacrylamide gel electrophoresis and subsequently transferred onto polyvinylidene difluoride membranes (Millipore, Bedford, MA, USA). These membranes were incubated overnight at 4°C with primary antibodies against FABP4 (12802-1-AP; Proteintech, Rosemont, IL, USA), VDR (#12550; Cell Signaling Technology, Danvers, MA, USA), UCP1 (sc-518171; Santa Cruz Biotechnology, Inc., Dallas, TX. USA), CIDEA (13170-1-AP; Proteintech), PPAR*γ* (#2435; Cell Signaling Technology), PPAR*α* (15540-1-AP; Proteintech), FGF21 (ab171941; Abcam, Cambridge, UK) and α/β-tubulin (#2148, Cell Signaling Technology). The membranes were then washed and incubated with horseradish peroxidase–labeled secondary antibodies for 1 h at room temperature. Immunoreactivity was determined using Immobilon-P (Millipore) or Chemi-Lumi One L (Nacalai Tesque), and chemiluminescence was visualized using Amasham Imager 680 (GE Healthcare, Chicago, IL, USA).

### Oil Red O staining

Cells were washed with phosphate-buffered saline, fixed with 4% paraformaldehyde, and stained with Oil Red O (Merck Millipore, Billerica, MA, USA). Images were captured using a BZX-700 microscope (Keyence, Osaka, Japan). To quantify lipid levels, the stain was dissolved in isopropyl alcohol, and its absorbance was measured at 490 nm using an iMark microplate absorbance reader (Bio-Rad Laboratories, Hercules, CA, USA).

### Cell viability

The viability of pre-differentiated and differentiated C3H10T1/2 cells was measured using the Cell Counting Kit-8 (Dojindo Molecular Technologies, Inc., Kumamoto, Japan) according to the manufacturer’s instructions. In addition, the absorbance at 570 nm was measured using an iMark microplate absorbance reader (Bio-Rad Laboratories).

### Reporter assay

Reporter assays were performed as previously described (Kusudo et al., 2019). C3H10T1/2 cells were transfected with pGL4.21-UCP1 and pRL-TK. In addition, 24 h later, cells were stimulated with 100 pM 1,25(OH)_2_D_3_ for 24 h. Cells were lysed, and the luciferase activity was measured using the Dual-Luciferase System (Promega, Madison, WI, USA).

### Quantification of relative mitochondrial copy number

C3H10T1/2 cells were differentiated for eight days. Total DNA was isolated using the NucleoSpin DNA RapidLyse (Takara Bio, Kyoto, Japan) according to the manufacturer’s instructions. To calculate relative mitochondrial DNA copy number, the expression of mitochondrially encoded cytochrome C oxidase I (*MT-CO1*) and NADH dehydrogenase [ubiquinone] flavoprotein 1 (*Ndfvu1*) genes was quantified using qRT-PCR (Amthor et al., 2007).

### Statistical analysis

Data are expressed as mean ± standard error of the mean. The number of samples was three to six for RT-qPCR and four for Oil Red O staining, cell viability assay, and the reporter assay. The significance of differences between two groups was assessed using Student’s *t*-test. The significance of differences between multiple groups was assessed using a one-way analysis of variance followed by Dunnett’s *post hoc* test. Statistical significance was set at *p* < 0.05.

## Results

### VDR is required for brown adipogenesis of C3H10T1/2 cells

**Figure 1:**
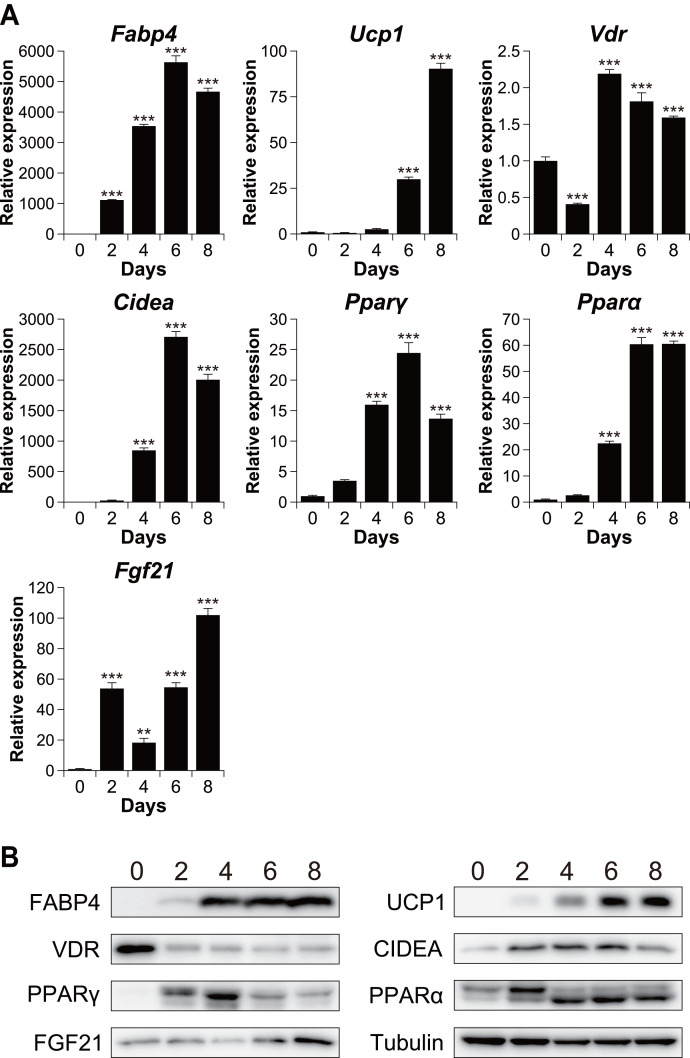
Time course analysis of mRNA and protein expression during brown adipocyte differentiation of C3H10T1/2 cells. (A) mRNA expression of *Fabp4, Ucp1*, *Vdr, Cidea, Pparγ*, * Pparα,* and *Fgf21*, as measured using quantitative reverse transcription-polymerase chain reaction (*n* = 4). (B) Protein expression of FABP4, UCP1, VDR, CIDEA, PPAR*γ*, PPAR*α*, FGF21, and α/β-tubulin, as measured using western blot analysis. Data are shown as mean ± standard error of the mean. ** *p* < 0.01, *** *p* < 0.001 *versus* day zero.

To investigate the relationship between vitamin D signaling and brown adipocyte differentiation, we first examined the expression of VDR in C3H10T1/2 cells, an established model of brown adipogenesis (Brunmeir et al., 2016) (Fig. 1A). After differentiation, the mRNA expression of the adipogenic markers fatty acid-binding protein 4 (*Fabp4*) and peroxisome proliferator-activated receptor *γ* (*Pparγ*) and that of the brown adipogenesis-related genes uncoupling protein 1 (*Ucp1*), cell death-inducing DFFA-like effector A (*Cidea*)*, Pparα*, and fibroblast growth factor 21 (*Fgf21*) significantly increased (*p* < 0.001). The expression of these genes was confirmed at the protein level (Fig. 1B). The mRNA expression of *Vdr* decreased transiently after stimulation (day 2) but recovered on day 4 and thereafter. However, at the protein level, VDR expression decreased after the differentiation (Fig. 1B).

**Figure 2:**
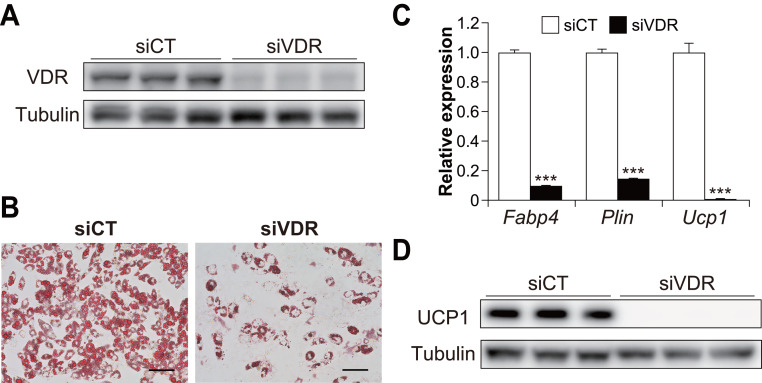
Vitamin D receptor (VDR) knockdown attenuated the brown adipocyte differentiation of C3H10T1/2 cells. C3H10T1/2 cells treated with small-interfering RNA (siRNA) were differentiated into mature brown adipocytes. (A) Western blot analysis of VDR and α/β-tubulin on day zero. (B) Representative images of Oil Red O staining in C3H10T1/2 cells after differentiation. Scale bars, 100 µm. (C) mRNA levels of *Fabp4*, *Plin*, and *Ucp1* after differentiation of C3H10T1/2 cells (*n* = 3). (D) Western blot analysis of UCP1 and α/β-tubulin on day 7. Representative images with three samples of each siRNA are shown. Data are shown as mean ± standard error of the mean. *** *p* < 0.001.

To examine the contribution of VDR to brown adipogenesis in C3H10T1/2 cells, we performed knockdown experiments using siRNA for *Vdr* in C3H10T1/2 cells (Fig. 2A). VDR knockdown significantly inhibited the differentiation of C3H10T1/2 cells (Fig. 2B). In addition, the expression of *Ucp1* and adipogenic markers *Fabp4* and perilipin (*Plin*) were significantly reduced in VDR knockdown cells compared to the control cells (all *p* < 0.001, Fig. 2C). Decreased UCP1 expression was also confirmed at the protein level (Fig. 2D). These results suggest that VDR plays a vital role in brown adipocyte differentiation.

### Effect of 1,25(OH)_**2**_D_**3**_ concentration on brown adipocyte differentiation

**Figure 3:**
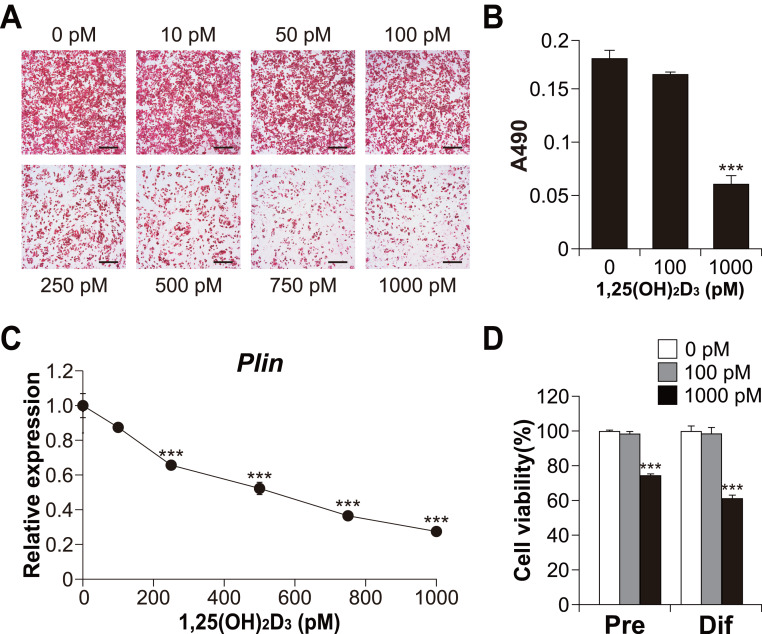
Effects of 1,25(OH)_2_D_3_ on the differentiation and viability of C3H10T1/2 cells. (A) Images of Oil Red O staining in differentiated C3H10T1/2 cells (scale bars, 500 µm) and (B) associated quantitative analysis of the extracted signal by spectrophotometry (*n* = 4). (C) Dose-dependent effects of 1,25(OH)_2_D_3_ on *Plin* mRNA expression (*n* = 3). (D) Effect of 1,25(OH)_2_D_3_ on C3H10T1/2 viability. Pre-differentiated (Pre) or differentiated (Dif) C3H10T1/2 cells were treated with 100 pM or 1000 pM 1,25(OH)_2_D_3_ for 24 h (*n* = 4). Data are shown as mean ± standard error of the mean. *** *p* < 0.001 versus control (0 pM).

To explore the role of vitamin D signaling in brown adipocyte differentiation, we investigated the effects of 1,25(OH)_2_D_3_ on brown adipocyte differentiation in C3H10T1/2 cells. As shown in Figs. 3A and 3B, high concentrations of 1,25(OH)_2_D_3_ suppressed lipid accumulation in the C3H10T1/2 cells cultured in adipogenic medium. In addition, consistent with Oil Red O staining, the expression of *Plin* (adipogenic and lipid droplet accumulation marker) decreased depending on the concentration of 1,25(OH)_2_D_3_ (Fig. 3C). Figure 3D shows the cytotoxic effect of 1,25(OH)_2_D_3_ on pre-differentiated or differentiated C3H10T1/2 cells. While 100 pM 1,25(OH)_2_D_3_ did not exert cytotoxic effects on either stage of C3H10T1/2 cells, 1,000 pM 1,25(OH)_2_D_3_ showed significant cytotoxic effects on both cell types (*p* < 0.001).

**Figure 4:**
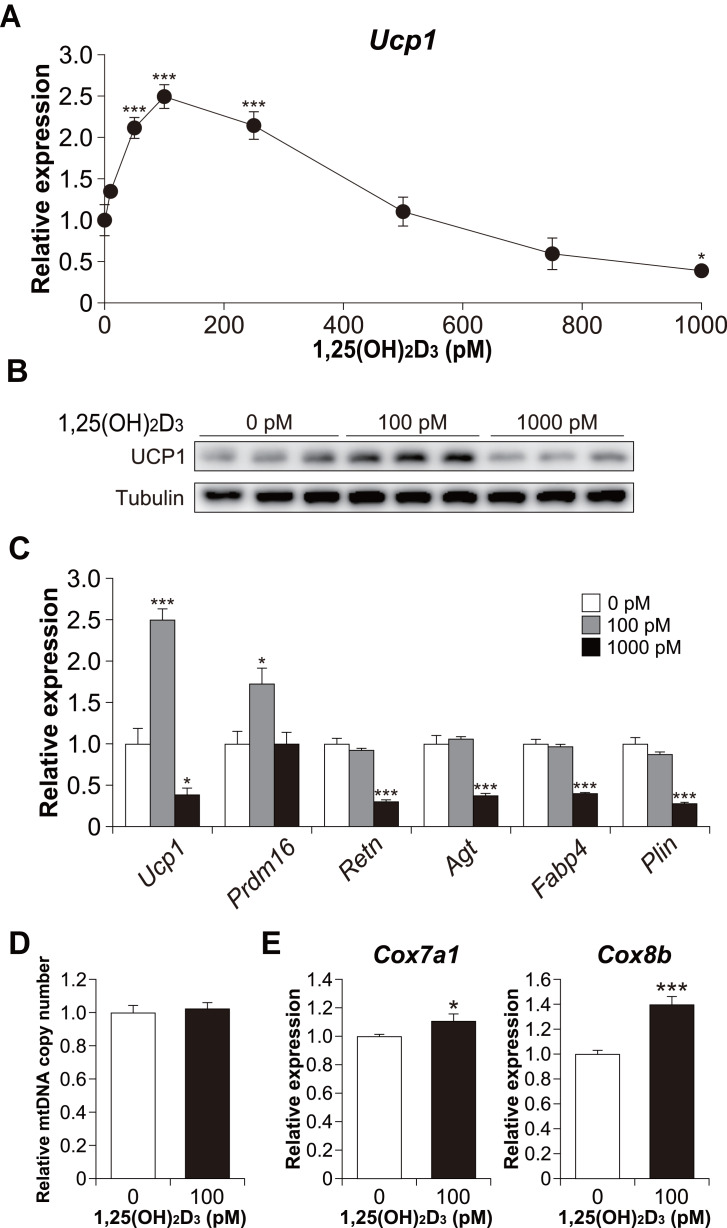
Effects of 1,25(OH)_2_D_3_ on brown adipocyte differentiation of C3H10T1/2 cells. (A) Dose-dependent effects of 1,25(OH)_2_D_3_ on *Ucp1* mRNA expression (*n* = 3). (B) UCP1 protein expression in differentiated C3H10T1/2 cells treated with 1,25(OH)_2_D_3_. (C) mRNA levels of brown fat-related (*Ucp1* and *Prdm16*), white fat-related (*Retn* and *Agt*), and adipocyte maker genes (*Fabp4* and *Plin*) (*n* = 3). (D) Relative mitochondrial (mt) DNA copy number (*n* = 6). (E) Expression of mitochondrial marker genes (*n* = 6). Data are shown as mean ± standard error of the mean. * *p* < 0.05, *** *p* < 0.001 versus control (0 pM).

Furthermore, we examined *Ucp1* mRNA expression in 1,25(OH)_2_D_3_-treated cells. Similar to the lipid accumulation results, at a high concentration of 1,25(OH)_2_D_3_, *Ucp1* expression decreased in a dose-dependent manner (Fig. 4A). However, *Ucp1* mRNA expression increased at low concentrations of 1,25(OH)_2_D_3_ (50–250 pM). UCP1 expression was confirmed at the protein level (Fig. 4B). Analysis of the effects of high (1,000 pM) and low (100 pM) concentrations of 1,25(OH)_2_D_3_ on the expression of different adipocyte differentiation markers revealed that the PR domain containing 16 (*Prdm16*), a master regulator of BAT differentiation, was significantly elevated in cells treated with 100 pM 1,25(OH)_2_D_3_ when compared with control cells (*p* = 0.030, Fig. 4C). Notably, 100 pM 1,25(OH)_2_D_3_ did not affect the expression of the white adipocyte-specific markers resistin (*Retn*) and angiotensinogen (*Agt*) or the adipogenic genes *Fabp4* and *Plin* when compared with the control cells. Except for *Prdm16*, the expression of all these genes was suppressed following treatment with 1,000 pM 1,25(OH)_2_D_3_ (*p* < 0.001), suggesting that a high concentration of 1,25(OH)_2_D_3_ inhibits the differentiation of C3H10T1/2 cells into brown and white adipocytes. At 100 pM, 1,25(OH)_2_D_3_ did not increase mitochondrial copy number (Fig. 4D) but significantly increased the mRNA expression of cytochrome c oxidase polypeptide (*Cox*)*7a1* (*p* = 0.05) and *Cox8b* (*p* <0.001), which are brown fat-selective mitochondrial genes (Fig. 4E). The blood concentration of 1,25(OH)_2_D_3_ is typically within 2–350 pM (Ryan et al., 2013), suggesting that a low concentration of 1,25(OH)_2_D_3_ stimulates the brown adipogenic program at physiological concentrations.

### 1,25(OH)_**2**_D_**3**_ affects the differentiation phase of brown adipogenesis

**Figure 5:**
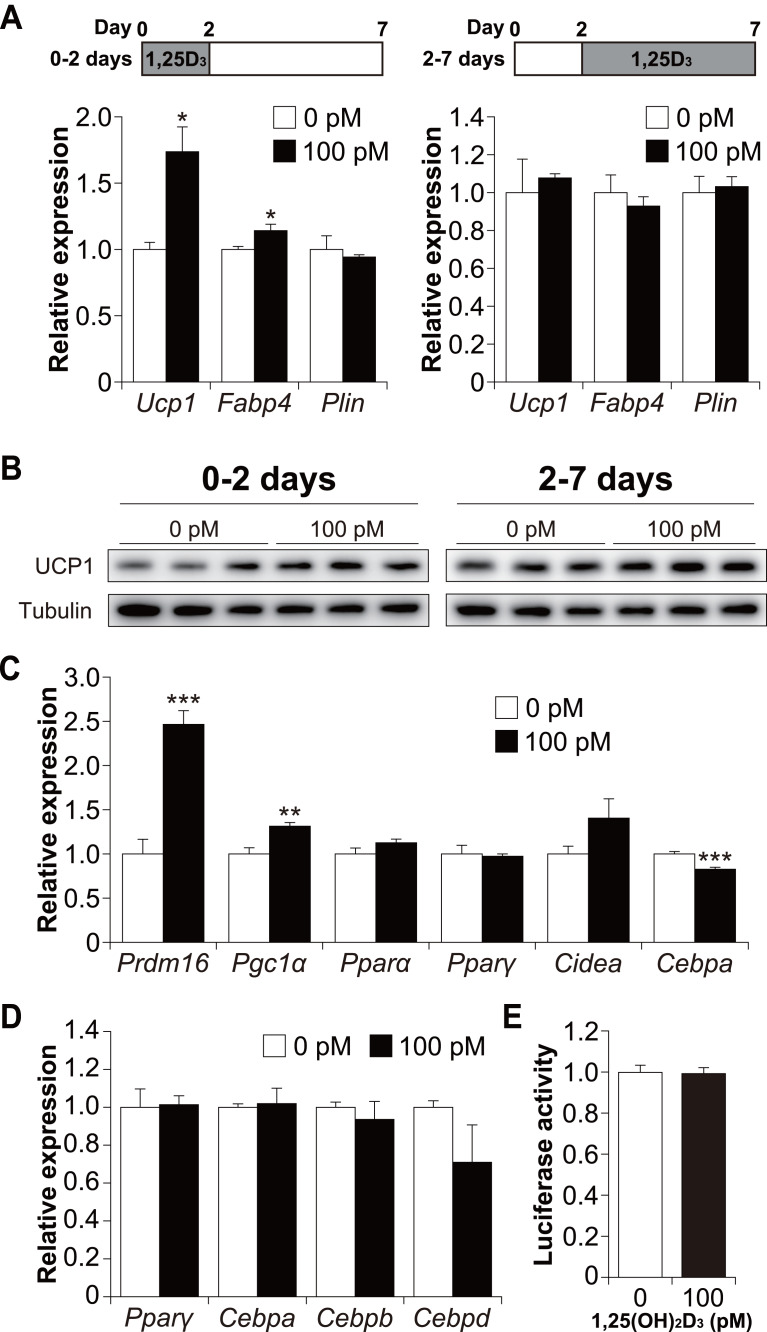
1,25(OH)_2_D_3_ acts during the early stages of brown adipogenesis. C3H10T1/2 cells were differentiated for 7 days; 100 pM 1,25(OH)_2_D_3_ or the vehicle control (EtOH) was added from day zero to 2 or from days 2 to 7. (A) mRNA levels of *Ucp1, Fabp4,* and *Plin* on day 7 (0–2 days; *n* = 3, 2–7 days; *n* = 4). 1,25(OH)_2_D_3_ (1,25D_3_) (B) UCP1 protein expression on day 7. (C) Expression of brown adipogenesis-related transcription factors. C3H10T1/2 cells were differentiated in the absence or presence of 100 pM 1,25(OH)_2_D_3_. mRNA levels of *Prdm16*, *Pgc1α*, *Pparα*, *Pparγ*, *Cidea*, and *Cebpa* were measured 48 h after the initiation of differentiation (*n* = 4). (D) Expression of adipogenesis-related transcription factors during the early stages of differentiation. C3H10T1/2 cells were differentiated in the absence or presence of 100 pM 1,25(OH)_2_D_3_ 12 h after differentiation initiation, and the mRNA levels of *Pparγ*, *Cebpa*, *Cebpb*, and *Cebpd* were measured using quantitative reverse transcription-polymerase chain reaction (*n* = 3). (E) Effect of 1,25(OH)_2_D_3_ on the stimulation of* Ucp1* promoter activity (*n* = 4). Data are shown as mean ± standard error of the mean. * *p* < 0.05, ** *p* < 0.01, *** *p* < 0.001 versus control (0 pM).

To determine whether 1,25(OH)_2_D_3_ affects the early or late phase of brown adipogenesis, C3H10T1/2 cells were treated with 1,25(OH)_2_D_3_ specifically during the differentiation phase (first 48 h) and cultured without 1,25(OH)_2_D_3_ (days 2 to 7). As shown in Fig. 5A, treatment with 100 pM 1,25(OH)_2_D_3_ for the first two days increased *Ucp1* and *Fabp4* mRNA expression in C3H10T1/2 cells compared to that in the control cells (*p* = 0.019 and *p* = 0.049, respectively). *Plin* expression, however, remained unchanged. We then investigated the effect of 1,25(OH)_2_D_3_ on the maturation phase of brown adipogenesis (days 2 to 7). To achieve this, C3H10T1/2 cells were initially differentiated in the absence of 1,25(OH)_2_D_3_ and then treated with 100 pM 1,25(OH)_2_D_3_ from days 2 to 7. The addition of 1,25(OH)_2_D_3_ did not affect the expression of *Ucp1*, *Fabp4*, or *Plin*. UCP1 protein expression under both conditions showed the same tendency as the mRNA expression (Fig. 5B). Therefore, a physiological concentration of 1,25(OH)_2_D_3_ enhanced brown adipocyte differentiation of C3H10T1/2 cells by acting during the early stages of brown adipogenesis. To further understand the molecular mechanism by which 1,25(OH)_2_D_3_ stimulates the brown adipocyte differentiational program, we examined the expression of transcription factors 48 h after induction of differentiation. As shown in Fig. 5C, 1,25(OH)_2_D_3_ treatment significantly increased the mRNA expression of *Prdm16* and peroxisome proliferator-activated receptor *γ* coactivator-1*α* (*Pgc1α*) and slightly decreased the expression of CCAAT-enhancer-binding protein *α* (*Cebpa*) (*p* < 0.001, *p* = 0.003, and *p* < 0.001, respectively). *Pparα*, *Pparγ*, and *Cidea* were unaffected by 1,25(OH)_2_D_3_ treatment. Studies have reported that at an early stage of differentiation, the CEBP family and PPAR*γ* play an essential role in brown and white adipogenesis (Wu, Bucher & Farmer, 1996; Yubero et al., 1994; Manchado et al., 1994). Therefore, we further investigated the expression levels of *Pparγ*, *Cebpa*, *Cebpb*, and *Cebpd* 12 h after the initiation of differentiation. However, the addition of 1,25(OH)_2_D_3_ did not alter the mRNA expression of these genes (Fig. 5D). Malloy & Feldman (2013) reported that VDR directly modulates UCP1 expression. The effect of 1,25(OH)_2_D_3_ on *Ucp1* promoter activity was examined. As shown in Fig. 5E, *Ucp1* promoter activity was not affected by adding 1,25(OH)_2_D_3_.

### 1,25(OH)_**2**_D_**3**_ inhibits white adipocyte differentiation in a dose-dependent manner but enhances *Ucp1* expression in 3T3-L1 beige adipogenesis

**Figure 6:**
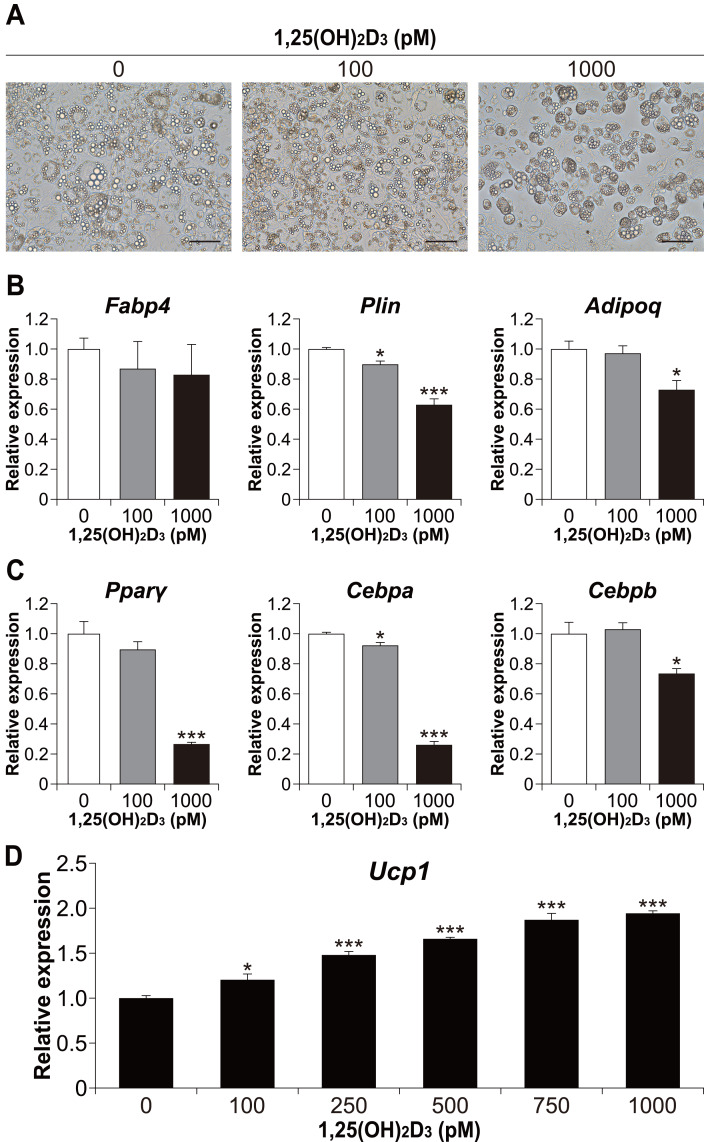
1,25(OH)_2_D_3_ inhibits white adipogenesis in 3T3-L1 cells but promotes beige adipocyte differentiation in 3T3-L1 cells. 3T3-L1 cells were differentiated with 0 pM (EtOH), 100 pM, or 1000 pM 1,25(OH)_2_D_3_. (A) Representative images of cell culture after differentiation on day eight. Scale bars, 100 µm. (B) mRNA levels of *Fabp4*, *Plin*, and *Adipoq* on day six (*n* = 4). (C) Expression of adipogenesis-related transcription factors. 3T3-L1 cells were differentiated in the absence or presence of 1,25(OH)_2_D_3_. mRNA levels of *Pparγ*, *Cebpa*, and *Cebpb* were measured 48 h after the initiation of differentiation (*n* = 4). (D) 3T3-L1 cells differentiated for eight days under browning conditions supplemented with various concentration of 1,25(OH)_2_D_3_. mRNA expression of *Ucp1* was measured by real-time reverse transcription polymerase chain reaction (*n* = 4). Data are shown as mean ± standard error of the mean. * *p* < 0.05, *** *p* < 0.001 *versus* 0 pM.

We also examined whether the effect of 1,25(OH)_2_D_3_ on differentiation in the physiological range is specific to brown adipocyte differentiation. To investigate the effect of 1,25(OH)_2_D_3_ on white adipocyte differentiation, 3T3-L1 cells, a widely used white adipocyte differentiation model, were differentiated with 1,25(OH)_2_D_3_. As shown in Fig. 6A, treatment with 1,000 pM 1,25(OH)_2_D_3_ inhibited the differentiation of 3T3-L1 cells. At 100 pM, there was no apparent morphological difference in 3T3-L1 cells compared to that in the control, but the *Plin* mRNA level was slightly decreased (*p* = 0.025, Fig. 6B). In addition, the expression of *Plin* and adiponectin (*Adipoq*) was significantly reduced at 1,000 pM 1,25(OH)_2_D_3_ (*p* < 0.001 and *p* = 0.011, respectively). Thus, 1,25(OH)_2_D_3_ inhibits white adipocyte differentiation in a dose-dependent manner. To investigate the inhibitory effect of 1,25(OH)_2_D_3_ on white adipogenesis of 3T3-L1, we measured the expression of *Pparγ*, *Cebpa*, and *Cebpb* after 48 h of initiation of differentiation. At 1,000 pM, 1,25(OH)_2_D_3_ significantly downregulated these genes (*p* < 0.001, *p* < 0.001, and *p* = 0.010, respectively, Fig. 6C), which suggested that the inhibitory effect of 1,25(OH)_2_D_3_ on 3T3-L1 differentiation is exerted through suppression of regulator gene expression. 3T3-L1 cells were reported to differentiate into beige adipocytes with long-term treatment with rosiglitazone, T3, and IBMX (Asano et al., 2014). Therefore, we verified the effect of 1,25(OH)_2_D_3_ on beige adipogenesis of 3T3-L1. As shown in Fig. 6D, 1,25(OH)_2_D_3_ significantly increased *Ucp1* mRNA expression in a dose-dependent manner at 100 pM (*p* = 0.011) or higher (*p* < 0.001).

## Discussion

In this study, we investigated the effects of 1,25(OH)_2_D_3_ on brown adipocyte differentiation of C3H10T1/2 cells. We found that 1,25(OH)_2_D_3_ has a biphasic effect on this process, with physiological concentrations enhancing differentiation and high concentrations inhibiting differentiation.

In a previous study, Ricciardi et al. (2015) reported that 1,25(OH)_2_D_3_ inhibited brown adipocyte differentiation and mitochondrial respiration in a dose-dependent manner (Ricciardi et al., 2015). The discrepancy between their data and the present study is probably the differences in the concentration of 1,25(OH)_2_D_3_ and cell types used in both studies. The 1,25(OH)_2_D_3_ concentration used in their study was greater than 1 nM, which is very high compared to the physiological concentration. Although the working concentrations in our experiment differed from those in their study, 1,25(OH)_2_D_3_ also inhibited brown adipogenesis of C3H10T1/2 cells in the high concentration range. However, at a physiologically relevant concentration of 100 pM (*i.e.,* within the 2–350 pM serum concentration range), 1,25(OH)_2_D_3_ positively stimulated brown adipogenesis.

Several studies have reported that 1,25(OH)_2_D_3_ exerts an inhibitory effect on white adipocyte differentiation (Kelly & Gimble, 1998; Blumberg et al., 2006; Kong & Li, 2006; Basoli et al., 2017; Zhuang, Lin & Yang, 2007; Sakuma et al., 2012). Similarly, 1,25(OH)_2_D_3_ suppressed 3T3-L1 differentiation in our results at both 100 pM and 1000 pM concentrations. This suggests that 1,25(OH)_2_D_3_ exerts a suppressive effect on white adipocyte differentiation and has distinct effects on brown and white adipogenesis. Furthermore, Kong & Li (2006) reported that 1,25(OH)_2_D_3_ blocks the adipogenic program in 3T3-L1 cells by suppressing the expression of *Cebpa* and *Pparγ*, the master regulators of adipogenesis. This result was confirmed in our study (Fig. 6C). In our experiments using C3H10T1/2 cells, 1,25(OH)_2_D_3_ treatment slightly decreased *Cebpa* mRNA expression but did not stimulate the expression of *Pparγ*. However, we observed a significant increase in the expression of *Prdm16* and *Pgc1α* in cells treated with 100 pM 1,25(OH)_2_D_3_, which suggested that 1,25(OH)_2_D_3_ stimulates brown adipogenesis *via Prdm16* and *Pgc1α* upregulation. Further studies are required to clarify the precise mechanism by which 1,25(OH)_2_D_3_ increases *Prdm16* and *Pgc1α* expression.

The role of the vitamin D system in energy metabolism has been explored in VDR knockout mice (Wong et al., 2009; Narvaez et al., 2009). These mice demonstrated lower body fat mass, higher energy expenditure, and increased UCP1 expression in WAT. These results suggest a suppressive role for vitamin D signaling in BAT generation and, therefore, seem to contradict our findings. However, VDR knockout mice also show a lean and alopecia-like phenotype (Sakai & Demay, 2000; Li et al., 1997). The thermoneutral temperature for mice is approximately 30°C; therefore, normal housing conditions (18–23°C) impose chronic thermal stress on these animals (Feldmann et al., 2009; Cannon & Nedergaard, 2011). The increase in body surface area and hair loss may make VDR knockout mice feel colder than the control mice. The sympathetic nervous system, the most important stimulus for adipose tissue browning, may demonstrate elevated activity in VDR knockout mice compared with that in the control mice. Consequently, increased energy expenditure and UCP1 expression in WAT may represent compensatory effects associated with a cold environment rather than a direct effect of the vitamin D system. Experiments conducted under thermoneutral conditions are needed to clarify the role of the vitamin D system in brown fat generation and energy expenditure. High energy expenditure, as seen in VDR knockout mice, is contrary to the findings of the majority of human studies that show an inverse correlation between adiposity/obesity and vitamin D status (Bell et al., 1985; Snijder et al., 2005). Our results support the findings of those human epidemiological and clinical studies.

## Conclusions

In the brown adipogenesis of C3H10T1/2 cells, a high concentration of 1,25(OH)_2_D_3_ inhibits differentiation; however, the physiological concentration of 1,25(OH)_2_D_3_ upregulates certain brown adipose markers such as *Ucp1*, *Prdm16*, and *Pgc1α*. In addition, *Ucp1* expression was induced by 1,25(OH)_2_D_3_ in the beige adipogenesis of 3T3-L1 cells. Further studies should be performed to clarify the underlying molecular mechanism and demonstrate the effect *in vivo*. Nevertheless, our findings suggest that supplementation of vitamin D and its analogs may represent an effective therapeutic strategy for the treatment of obesity and related metabolic diseases through the promotion and inhibition of brown and white adipocyte differentiation, respectively.

##  Supplemental Information

10.7717/peerj.14785/supp-1 Supplemental Information 1 Raw data

10.7717/peerj.14785/supp-2 Supplemental Information 2 WB uncropped blots

## Additional Information and Declarations

### Data Availability

The following information was supplied regarding data availability: The raw measurements are available in the Supplemental Files.
