# Supplementary figures and images for: Bidirectional effect of vitamin D on brown adipogenesis of C3H10T1/2 fibroblast-like cells
Source: PeerJ. 2023 Jan 31;11:e14785. doi: 10.7717/peerj.14785 (PMC9934812; doi:10.7717/peerj.14785)

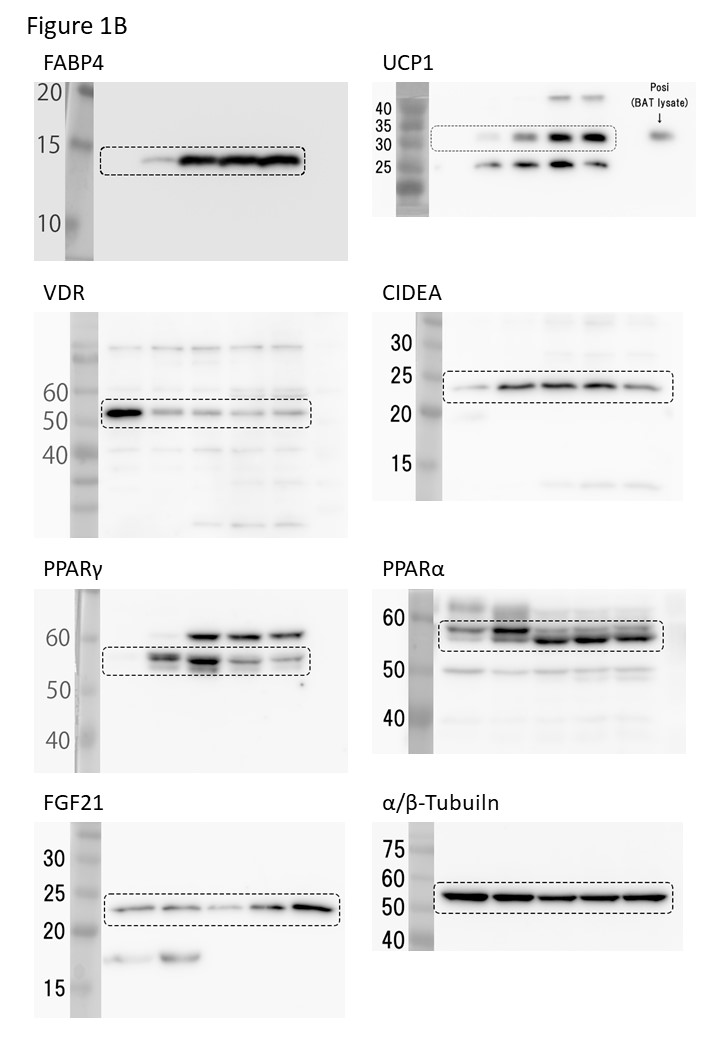

Supplement: Supplemental Information 2 [file peerj-11-14785-s002.zip › Figure1B_WB_uncropped_data.jpg]

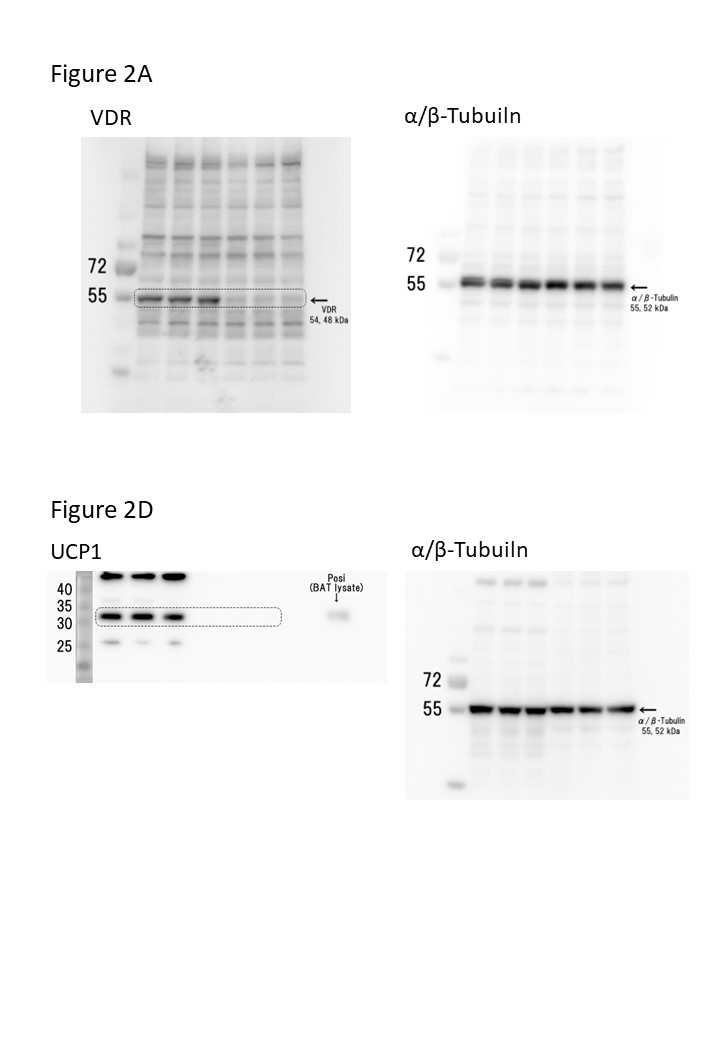

Supplement: Supplemental Information 2 [file peerj-11-14785-s002.zip › Figure2A_2D_WB_uncropped_data.jpg]

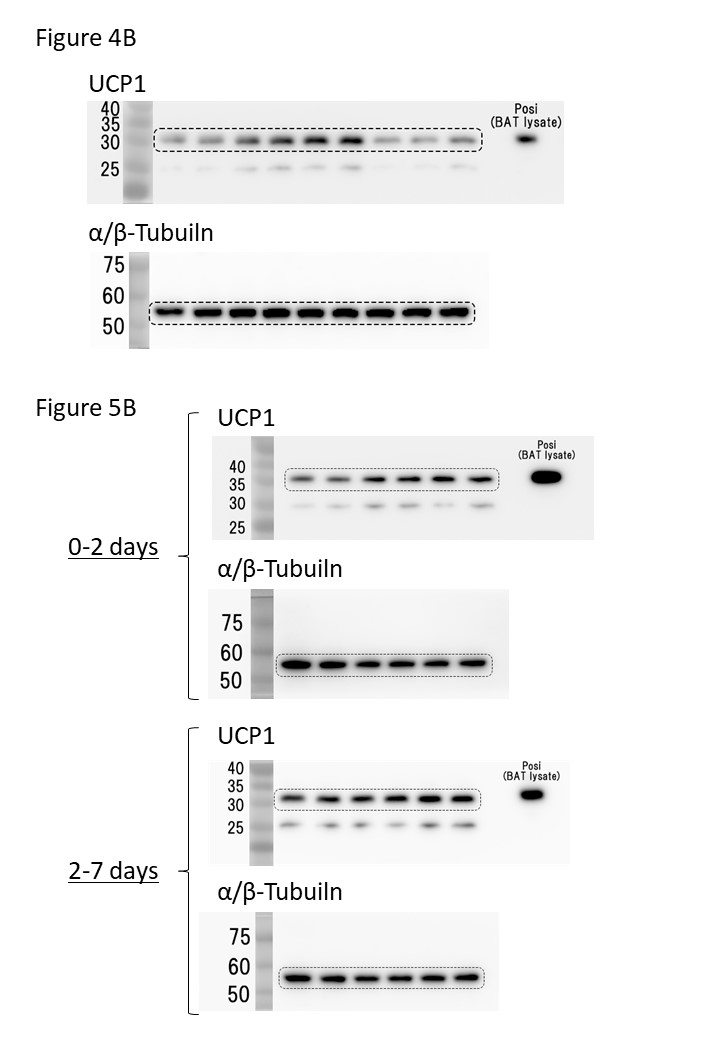

Supplement: Supplemental Information 2 [file peerj-11-14785-s002.zip › Figure4B_5B_WB_uncropped_data.jpg]
